# Integrated Process for Ethanol, Biogas, and Edible Filamentous Fungi-Based Animal Feed Production from Dilute Phosphoric Acid-Pretreated Wheat Straw

**DOI:** 10.1007/s12010-017-2525-1

**Published:** 2017-06-08

**Authors:** Ramkumar B. Nair, Maryam M. Kabir, Patrik R. Lennartsson, Mohammad J. Taherzadeh, Ilona Sárvári Horváth

**Affiliations:** Swedish Centre for Resource Recovery, University of Borås, 50190 Borås, SE Sweden

**Keywords:** Wheat straw, Dilute acid pretreatment, Filamentous fungi, Bioethanol, Biogas, Integration, *N. intermedia*

## Abstract

Integration of wheat straw for a biorefinery-based energy generation process by producing ethanol and biogas together with the production of high-protein fungal biomass (suitable for feed application) was the main focus of the present study. An edible ascomycete fungal strain *Neurospora intermedia* was used for the ethanol fermentation and subsequent biomass production from dilute phosphoric acid (0.7 to 1.2% *w*/*v*) pretreated wheat straw. At optimum pretreatment conditions, an ethanol yield of 84 to 90% of the theoretical maximum, based on glucan content of substrate straw, was observed from fungal fermentation post the enzymatic hydrolysis process. The biogas production from the pretreated straw slurry showed an improved methane yield potential up to 162% increase, as compared to that of the untreated straw. Additional biogas production, using the syrup, a waste stream obtained post the ethanol fermentation, resulted in a combined total energy output of 15.8 MJ/kg wheat straw. Moreover, using thin stillage (a waste stream from the first-generation wheat-based ethanol process) as a co-substrate to the biogas process resulted in an additional increase by about 14 to 27% in the total energy output as compared to using only wheat straw-based substrates.

**Figure:**
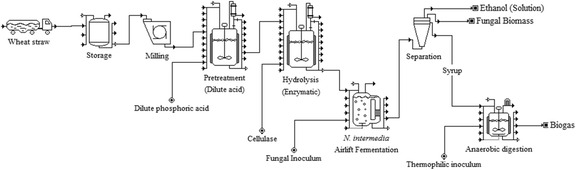
Graphical Abstract

Graphical Abstract

## Introduction

There are currently several technologies for ethanol from lignocelluloses that are successfully approved in pilot and demo plants. However, their economical aspects for successful commercial production are still a challenging issue. For the lignocellulose-based process to be economical, the concept of a “*biorefinery*” that combines the production of various biofuels, chemicals, heat or electricity, etc. using lignocellulosic waste materials has to be considered [1–3]. The concept of *biorefinery* can make use of the additional or intermediate products produced, maximizing the value derived from the feedstock according to the economical or market situations and the biomass availability. Among the various potential lignocellulose feedstocks for a biorefinery approach, wheat straw is a low-cost and abundantly available agricultural residue. According to the International Grains Council, wheat (with a straw to grain ratio of 1.3) is the second largest grain globally produced, with an estimated annual production of 747 million tons (for the year 2016–2017) [4]. Presently, the process of bioethanol production from wheat straw, through microbial fermentation [5–7], or its conversion to biogas is studied extensively [8–10]. However, research based on an “*integrated biorefinery*” perspective, that focus on ethanol fermentation followed by biogas production, is not explored extensively. The wider availability of wheat straw makes it a suitable feedstock for biofuel production in the integrated biorefinery model. However, the recalcitrant nature of wheat straw (with its composition of 30–40% cellulose, 20–25% hemicellulose, and 20–25% lignin [11]) underscores the need for a pretreatment process prior to its sugar hydrolysis for biofuel production. In the integrated process, the choice of pretreatment is crucial as it is required for both ethanol and biogas processes. There have been many studies conducted previously on pretreatment of lignocellulosic biomass using different methods, of which the most common is the use of sulfur compounds (e.g., SO_2_ or sulfuric acid) as catalysts [12, 13]. The presence of sulfur, however, has proved to cause problems considering the biogas production in the integrated process [14]. The presence of sulfate stimulates the growth of sulfate-reducing bacteria which can compete with methanogens on the available substrate. Increased activity of sulfate reducers will lead to reduced methane yield and simultaneously to increased hydrogen sulfide content in the produced biogas. Higher concentrations of hydrogen sulfide deteriorate the gas quality and can cause challenges in pipelines and tanks due to its corrosive nature [15, 16]. A pretreatment process using dilute phosphoric acid has been previously introduced by Nair et al. [17], wherein the presence of residual phosphate in the hydrolysate media serves as a nutrient source for microbial activity [18], enhancing the ethanol yield. The presence of phosphorus has also been reported to enhance the overall biogas yield in the previous studies using corn stover as a biogas substrate [19]. Hence, in the present study, the dilute acid pretreatment using phosphoric acid is introduced for a combined ethanol and biogas process for the first time, with the pretreatment carried out in a demo-scale facility.

Considering the ethanol process from wheat straw, rendering the utilization of pentoses (C5 sugars) is a significant challenge, since *Saccharomyces cerevisiae*, the most robust ethanol-fermenting organism, in its wild type is unable to metabolize pentoses [20]. Considerable research has been made during the past years to develop genetically modified *S. cerevisiae* that co-ferment C6 sugars (hexoses) and C5 sugars to ethanol at high yields, but has several technical limitations. An alternative method of pentose utilization and ethanol fermentation is achieved with the use of filamentous fungi. Many of the filamentous fungi have also been traditionally recognized as the source of nutritious, highly palatable functional foods by many societies around the globe [21]. Hence, in the biorefinery model proposed in the present study, the ethanol fermentation of wheat straw is achieved by an ascomycete filamentous fungus, *Neurospora intermedia* (traditionally used for the preparation of an indigenous Indonesian food—*oncom*). The fungal biomass produced can be further utilized as a protein-rich animal or fish feed component [22]. Moreover, the fungal biomass produced could potentially contribute to the increasing global feed demand, reaching close to one billion tones with an estimated annual turnover of about USD 400 billion [23].

**Fig. 1:**
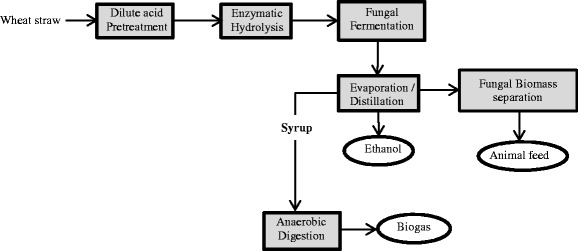
*Integrated biorefinery* approach using dilute phosphoric acid-pretreated wheat straw for ethanol, biogas, and edible fungi-based animal feed production

The present study proposes an integrated lignocellulosic biorefinery process with the use of wheat straw and edible filamentous fungi resulting in the production of ethanol, biogas, and fungi-based fish/animal feed. The paper describes (a) the evaluation of the wheat straw pretreatment using dilute phosphoric acid at a demonstration plant; (b) the filamentous fungal fermentation of the pretreated slurry post hydrolysis, carried out at laboratory pilot-scale airlift reactors for ethanol and fungal biomass production; and (c) the scope of improving biogas production potential from the fungal fermented wheat straw slurry, post the ethanol separation (Fig. 1). Furthermore, the methane production potential of thin stillage (waste stream from a wheat-based first-generation starch-to-ethanol facility) was also determined, considering its easy availability and with the objective of creating an economically flexible, entirely wheat-based integrated biorefinery model.

## Methods

### Materials

Commercial wheat straw (92.4% dry content) for both laboratory and demonstration experiments was supplied by Lantmännen Agroetanol (Norrköping, Sweden). Straw, with the composition (g/g, dry basis) arabinan 0.048 ± 0.013, galactan 0.0053 ± 0.0015, glucan 0.315 ± 0.061, mannan 0.0047 ± 0.0011, and xylan 0.24 ± 0.08, was milled (0.2–0.25 mm) using a laboratory rotor beater mill before use. Cellulase enzyme Cellic CTec2 (Novozymes, Denmark) with 94 filter paper unit (FPU)/mL activity [24] was used for the hydrolysis. Thin stillage, a residual product from the wheat-based first-generation ethanol facility, was provided by Lantmännen Agroetanol, Sweden. The thin stillage had a natural pH of 3.5, with the composition of total solids (% *w*/*v*) 9.2 ± 0.4 and suspended solids (% *w*/*v*) 2.2 ± 0.6 and (g/L) total nitrogen 4.8 ± 0.5, xylose 0.8 ± 0.1, arabinose 1.5 ± 0.1, glycerol 7.0 ± 0, lactic acid 1.8 ± 0.1, acetic acid 0.21 ± 0.01, and ethanol 1.2 ± 0.2.

### Fungal Strain

*N. intermedia* CBS 131.92 (Centraalbureau voor Schimmelcultures, Netherlands), an edible ascomycete fungus, was used for ethanol fermentation. The fungus was maintained on potato dextrose agar (PDA) plates containing (in g/L) potato extract 4, dextrose 20, and agar 15. Spore suspension was prepared by flooding the fungal plates with 20 mL sterile distilled water, and the spores were released by dispensing the mycelium with a disposable cell spreader. An inoculum of 30 mL spore suspension (with a spore concentration of 3–4 × 10^6^ spores/mL) per liter of the medium was used for the cultivations. For preparing fungal biomass inoculum, the spores were inoculated into 100 mL YEPD broth containing (in g/L) dextrose 20, peptone 20, and yeast extract 10. The culture was incubated for 48 h at 35 °C and 125 rpm. The fungal biomass was then harvested at the end of the cultivation and used as the inoculum, with its dry weight calculated by drying the fungal biomass at 105 °C overnight.

### Dilute Phosphoric Acid Pretreatment of Wheat Straw in the Demo Plant

Pretreatment of wheat straw (WS) was carried out at the Biorefinery Demo Plant (BDP), operated by SP (Technical Research Institute of Sweden) at Örnsköldsvik, Sweden. The reactions were performed in a 30-L one-step vertical plug flow continuous reactor with varying concentrations of acid continuously added in the feed screw prior to the reactor. Based on the preliminary laboratory study [25], two test runs were carried out at the demonstration plant at conditions (a) acid concentration 1.2% (*w*/*v*), residence time 7 min, and temperature 195 ± 2 °C with final slurry pH of 2.9 ± 0.1, designated hereafter as test run 1 (R1), and (b) acid concentration 0.7% (*w*/*v*), residence time 7 min, and temperature 201 ± 4 °C with final slurry pH of 3.4 ± 0.2, designated hereafter as test run 2 (R2). For each of the test runs, a total of 1000 L pretreated slurry was collected separately and stored.

### Enzymatic Hydrolysis and Fermentation: Comparison of SSF and SHF Process

A comparison between simultaneous saccharification and fermentation (SSF) and separate hydrolysis and fermentation (SHF) experiments was carried out using dilute phosphoric acid-pretreated straw from the demo plant. Enzymatic hydrolysis was initiated at the solid loading of 7.5%, pH 5.5 ± 0.1, and enzyme loading of 10 FPU/g dry substrate [25]. For SSF process, fungal spores (3.4 × 10^6^ spores/mL) and enzymes were simultaneously added to the shake flask (100 mL working volume) and incubated at 35 °C for 120 h. While for SHF process, the addition of fungal spores (3.9 × 10^6^ spores/mL) was achieved post the enzymatic hydrolysis for 48 h, and then, the fermentation was carried out for another 72 h. In both these processes, samples were collected every 24 h. Modified SHF experiments were also carried out to quantify the fungal biomass produced during the fermentation, wherein the solid particles from the enzymatically hydrolyzed (48 h) acid-pretreated straw were removed through centrifugation followed by vacuum filtration. The liquid hydrolysate (filtrate) was inoculated with fungal biomass (0.95 g dry biomass/L media), and then, the fermentation was carried out for another 72 h. The wet fungal biomass was harvested and dried at 105 °C overnight, to determine the biomass dry weight content.

### Scale-Up in an Airlift Bioreactor

The scale-up experiments (SSF) were carried out in a bench-scale airlift bioreactor (4.5 L) (Belach Bioteknik, Sweden), with a working volume of 3.5 L, by the simultaneous addition of enzyme (10 FPU/g substrate) and fungal biomass (12.5 ± 0.8 mg dry biomass/L media) with a final substrate loading of 7.5% (*w*/*w*) of dry matter content that was maintained. Airlift liquid circulation was achieved by an internal loop with cylindrical geometry with a diameter of 58 mm, height of 400 mm, and thickness of 3.2 mm and was used to achieve the airlift liquid circulation. Aeration at the rate of 1.0 volume_air_/volume_media_/min (vvm) was maintained throughout the cultivation, using a sintered stainless steel air sparger with a pore size of 90 μm. Filtration of inlet air was achieved by using a membrane filter (0.1-μm pore size, Whatman, Florham Park, NJ, USA). The cultivation was carried out at pH 5.5 ± 0.2 initially adjusted with 1 M NaOH. The fermentation was carried out at a temperature of 35 ± 2 °C for 120 h.

### Batch Anaerobic Digestion

Batch digestion assays were performed according to the method described by Hansen et al. [26] using thermophilic inoculum obtained from a large-scale digester treating municipal solid waste at 55 °C (Borås Energi och Miljö AB, Borås, Sweden). The digesters used were serum glass bottles with a total volume of 118 mL, closed with butyl rubber seals and aluminum caps. Each flask contained 40 mL of inoculum and 0.25 g volatile solids (VSs) of untreated wheat straw and pretreated straw from test R1 and test R2 (see Dilute Phosphoric Acid Pretreatment of Wheat Straw in the Demo Plant section) to achieve a VS ratio of inoculum to substrate of 2:1. The effluent from fungal fermentation (ethanol process in Enzymatic Hydrolysis and Fermentation: Comparison of SSF and SHF Process section) was subjected to rotary evaporation process for ethanol separation, preparing the syrup for the biogas production process which is designated hereafter as syrup R1 and syrup R2 (from test run 1 and test run 2, respectively). The loss of water during the evaporation process was compensated by distilled water addition. Batch anaerobic digestion of the syrup was also carried out at similar conditions to the untreated or pretreated straw samples. Considering the availability of thin stillage (a waste stream from the wheat-based first-generation process), the methane production from thin stillage alone, and its combination with syrup R1 and R2 (at the ratio of 1:1 based on the VS content), was also investigated. Furthermore, digester containing inoculum alone was used as blanks for the determination of the gas production of the inoculum itself. The headspace of each bottle was flushed with a mixture of 80% nitrogen and 20% carbon dioxide to obtain anaerobic conditions. Gas samples were withdrawn regularly from the headspace of each bottle, and the accumulated methane production was determined using gas chromatography.

### Analytical Methods

The total solid (TS), suspended solid (SS), volatile solid (VS), and total sugar content in the untreated and phosphoric acid-pretreated wheat straw biomass was measured according to the National Renewable Energy Laboratory (NREL) methods [27, 28]. High-performance liquid chromatography (Waters 2695, Waters Corporation, USA) was used to analyze the liquid fractions from the pretreated, enzyme hydrolyzed, and fermented wheat straw biomass. A hydrogen ion-based ion exchange column (Aminex HPX-87H, Bio-Rad Hercules, CA, USA) at 60 °C with a Micro-Guard Cation H guard column (Bio-Rad) and 0.6 mL/min 5 mM H_2_SO_4_ as eluent was used for the analyzes of glucose, ethanol, acetic acid, furfural, and 5-hydroxymethyl-furfural. On the other hand, for the separation of glucose, mannose, galactose, cellobiose, xylose, and arabinose, a lead (II)-based column (Aminex HPX-87P, Bio-Rad) at 85 °C with a set of two Micro-Guard Deashing, followed by a Micro-Guard Carbo-P guard column (Bio-Rad) and 0.6 mL/min ultrapure water as eluent, was used. Separation of ethanol from the fungal fermented slurry was achieved using a large-scale evaporator (Laborota 20, Heidolph, Schwabach, Germany). The methane production was measured using a gas chromatograph (Auto System PerkinElmer, Inc., Waltham, MA) equipped with a packed column (PerkinElmer, 60x1, 800OD, 80/100, Mesh) and a thermal conductivity detector (PerkinElmer) with an injection temperature of 150 °C. Nitrogen was used as carrier gas, with a flow rate of 23 mL/min at 60 °C. The gas sampling was performed with a 250-μL pressure-tight gas syringe (VICI, Precision Sampling Inc., LA). All methane volumes are presented under the standard conditions (temperature 273 K and pressure 101,325 Pa). Structural changes in the pretreated wheat straw were investigated using a Fourier transform infrared (FTIR) spectrometer (Impact 410 iS10, Nicolet Instrument Corp., Madison, WI, USA). The spectrum data was developed by Nicolet OMNIC 4.1 software (Nicolet Instrument Corp.) and analyzed by eFTIR (Essential FTIR, USA). The crystallinity index was calculated as the ratio of the absorbance at wavenumber 1420 and 898 cm^−1^ [29]. The viscosity of the pretreated slurry was determined using a Brookfield digital viscometer model DV-E (Chemical Instruments AB, Sweden).

## Results and Discussion

### Validation of Dilute Acid Pretreatment at the Biorefinery Demonstration Plant

The optimum pretreatment conditions obtained from the laboratory experiments were validated at the biorefinery demonstration plant. The addition of straw to the reactor was carried out continuously with a flow rate adjusted between 0.7 and 1.7 L/h. Limitations in maintaining the temperature were observed with the vertical reactor, especially in terms of the material output in the continuous process. The process was unstable at 190 °C in test R1, and the temperature was hence increased until stable conditions could be reached. Due to lower acid dosage, test R2 was performed at a higher temperature to achieve process stability. Choice of the high temperature being a critical factor for lignocellulose pretreatment at low acid dosage has been studied previously [30] especially with sulfuric acid as pretreatment catalyst [31, 32]. It was observed that in order to stabilize the reactor temperatures, size reduction of the material (straw) prior to pretreatment was optimal. However, the impact of these physical modifications of the biomass, including particle size reduction, on pretreatment performance is not well understood [33]. The pretreatment process produced about 1000 L pretreated straw slurry from each test run. The pretreated slurry R1 and R2 have the following characteristics: pH of 2.9 and 3.5, total solids (% *w*/*v*) 21.2 ± 0.1 and 23.2 ± 0.6, and suspended solids (% *w*/*v*) 14.4 ± 0.4 and 16.6 ± 0.9, respectively. The composition of solid and liquid fractions of the pretreated wheat straw obtained from the demo plant is depicted in Table 1. It was observed that an increase in acid concentration from 0.7 to 1.2% *w*/*v* resulted in an increase in total sugar release by about 81% as characterized from the straw hydrolyzate. However, about 57% increase in the total released inhibitor (furfural and HMF) concentrations was also observed. Nevertheless, in the present study, an improved pretreatment efficiency was observed, with about 96% increase of xylan hydrolysis yield (at pretreatment conditions 1.2% *w*/*v* acid and 7-min time) from wheat straw as compared to a previous study when using 1.75% *w*/*v* acid with a residence time of 10 min [25] at a similar demo-scale facility. The crystallinity index of the two pretreated straw samples was determined as 0.68 ± 0.02 and 0.49 ± 0.05, and the slurry had a viscosity of 92.1 ± 0.2 and 82.3 ± 0.6 cP after R1 and R2 treatment, respectively. The crystallinity index results are in accordance with the results obtained from the lab-scale pretreatment experiments, the value being 0.63 ± 0.03 at conditions 1.75% acid, 15 min, and 190 °C [25]. The decrease in the viscosity of the demo plant pretreated slurry compared to the lab results (108.5 ± 0.4 cP) could be attributed to the improved efficiency of the heat transfer and the mixing during the pretreatment at the demo plant [33].

**Table 1:** Chemical composition profile of dilute acid-pretreated wheat straw from the demonstration plant, treated at conditions acid conc. 1.2%, residence time 7 min, and temperature 195 ± 2 °C, i.e., R1, and acid conc. 0.7%, residence time 7 min, and temperature 201 ± 4 °C, i.e., R2

Component	Composition of the liquid fraction (g/L)		Composition of the solid fraction (g/g dry matter substrate)	
Sample	R1	R2	R1	R2
Arabinan	6.6 ± 0.2	5.04 ± 0.1	0.028 ± 0.001	0.031 ± 0.003
Glucan	4.6 ± 0.4	2.8 ± 0.2	0.246 ± 0.02	0.302 ± 0.010
Xylan	31.6 ± 0.1	15.8 ± 0.4	0.028 ± 0.007	0.016 ± 0.003
Acetic acid	2.8 ± 0.1	2.1 ± 0.0		
Furfural	5.6 ± 0.4	3.2 ± 0.3		
HMF	0.53 ± 0.02	0.39 ± 0.02		

### Enzymatic Hydrolysis and Fermentation

**Fig. 2:**
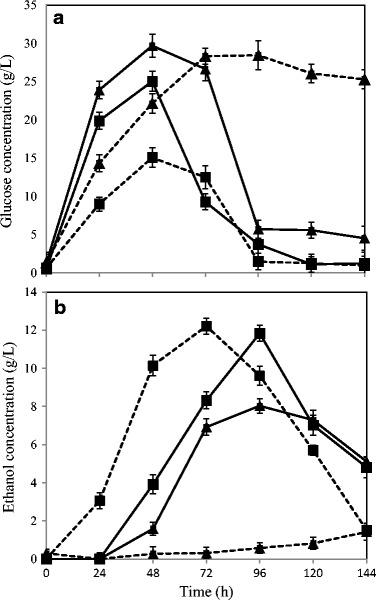
Fungal fermentation at separate hydrolysis and fermentation (*solid lines*) and simultaneous scarification and fermentation (*dotted lines*) process using dilute phosphoric acid-pretreated wheat straw at conditions acid conc. 1.2%, residence time 7 min, and temperature 195 ± 2 °C, i.e., R1 (represented as *triangles*), and acid conc. 0.7%, residence time 7 min, and temperature 201 ± 4 °C, i.e., R2 (represented as *squares*). The figure represents **a** glucose and **b** ethanol profile during 144 h of fermentation time

Both SSF and SHF processes were compared to determine the ethanol production efficiency at a reduced time and with the continuous assimilation of released fermentable sugars in the medium hence reducing the end-product inhibition [34]. A maximum ethanol concentration of 11.8 ± 0.5 g/L corresponding to 0.15 ± 0.005 g ethanol/g dry matter substrate straw, achieving 84% of the theoretical maximum based on the glucan content of the substrate, was obtained with the hydrolysate from R2 treatment, and after fermentation time of 120 h for the SHF process (Fig. 2). On the other hand, when using the same R2 hydrolysate in the SSF process, the maximum ethanol production time was reduced to 72 h with an improved final ethanol concentration of 12.2 ± 0.06 g/L corresponding to a yield of 0.16 ± 0.001 g ethanol/g dry matter of the substrate, wheat straw, and hence achieving 90% of the theoretical maximum yield calculated on the basis of the glucan content of the substrate, wheat straw. Thus in the present study, a similar ethanol yield was achieved without any nutrient supplementation to the wheat straw slurry as opposed to previous studies performed at similar conditions [25]. However, a reduced ethanol concentration, with a maximum of 1.42 ± 0.11 and 8.03 ± 0.02 g/L, was observed with the hydrolysate obtained after R1 pretreatment conditions during the fungal fermentation in both the SSF and the SHF process, respectively (Fig. 2). The cultivation of fungus in liquid hydrolysate (obtained after solid removal post the enzymatic hydrolysis of the pretreated straw) resulted in a maximum of 1.53 ± 0.04 and 3.71 ± 0.11 g/L dry fungal biomass for R1 and R2, respectively, while 0.94 ± 0.02 g/L dry fungal biomass was obtained from the untreated but enzymatically hydrolyzed straw. A similar fungal biomass yield of around 4 g/L was observed in previous studies with *N. intermedia* while using thin stillage (a waste stream from first-generation starch-based ethanol facilities) as the fermentation substrate [35].

**Fig. 3:**
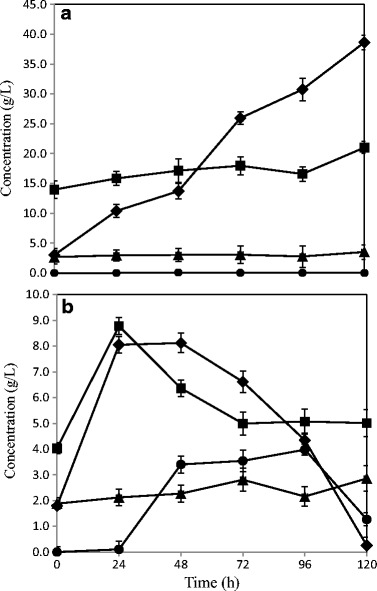
Sugar and metabolite profile during the SSF process of fungal fermentation in a bench-scale airlift bioreactors using dilute acid-pretreated wheat straw. **a** Condition R1. **b** Condition R2. The figure represents concentration (g/L) of ethanol (*circles*), total sugars (*squares*), glucose (*diamonds*), and acetic acid (*triangles*) for 120-h fermentation time

Scaling-up of the fermentation process was carried out in bench-scale airlift reactors in simultaneous hydrolysis and fermentation mode (SSF). Several studies have proved that the internal loop tube of the airlift promotes enhanced mixing pattern which leads to comparatively better mass and oxygen transfer rates, resulting in an improved fungal growth [36, 37]. The fermentation process reaching its maximum ethanol production at 96 h resulted in about 0.16 ± 0.03 g ethanol/g glucan content of untreated straw, while using wheat straw slurry R2 (Fig. 3). Previous studies using *N. intermedia* for thin stillage fermentation have reported an ethanol production of about 5 g/L, which would potentially lead to about 5.5% improvement in the overall process at the existing wheat-based first-generation ethanol facilities, producing 200,000 m^3^ ethanol per year [35]. However, no significant ethanol production was observed with hydrolysate R1, which could be accounted for the presence of an increased amount of inhibitors [25]. Nevertheless, a high glucose and sugar release, with about 0.41 ± 0.05 and 0.26 ± 0.13 g per g initial substrate, respectively, was observed with hydrolysate R1 and R2, during the airlift test, which could potentially be used for an improved methane yield during the subsequent biogas process [38].

### Biogas Production

**Fig. 4:**
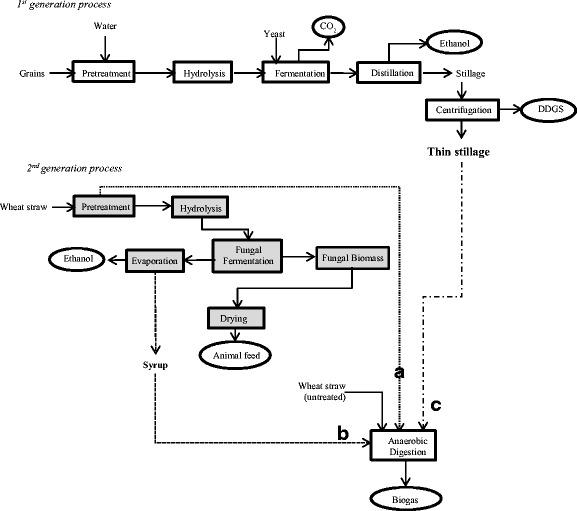
Process alternatives for the biogas production from wheat straw in the *integrated biorefinery* model. The figure explains three process scenarios for the integrated biogas production using **a** dilute phosphoric acid-pretreated wheat straw, **b** ethanol-separated fungal fermentation straw slurry (designated as syrup), and **c** thin stillage from first-generation starch-based ethanol process

**Fig. 5:**
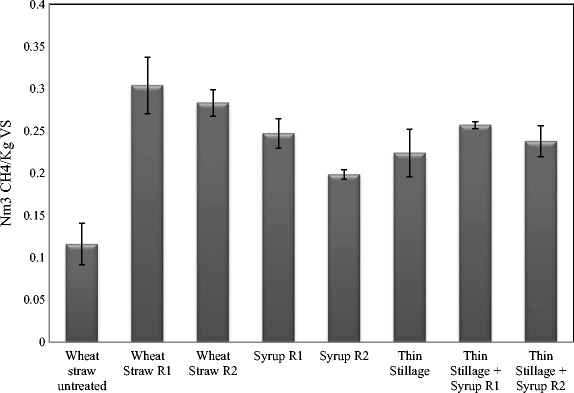
Accumulated methane yield (expressed as Nm^3^ CH_4_/kg VS) during 35 days of anaerobic digestion of various combinations of dilute phosphoric acid-pretreated and fungal fermented wheat straw (second-generation process) together with thin stillage from the first-generation ethanol facilities

Different waste streams collected from the various process alternatives (Fig. 4) were evaluated for biogas production in different combinations. The results of accumulated methane yields obtained after 35 days of digestion are shown in Fig. 5. The methane potential of untreated wheat straw was 0.12 ± 0.024 Nm^3^ CH_4_/kg VS, while the methane yields from the treated straw obtained after treatment conditions of R1 and R2 were 0.30 ± 0.033 and 0.28 ± 0.015 Nm^3^ CH_4_/kg VS, respectively (Fig. 4, process alternative a). Bauer et al. [39] have shown similar methane yield potential, with about 0.30–0.33 Nm^3^ CH_4_/kg VS produced from steam-pretreated (at 160–180 °C for 10–20 min) wheat straw in a combined ethanol and methane production process. The methane yield potential in the present study for the dilute acid treated straw was higher than those reported in previous studies [40] where 0.15 and 0.19 Nm^3^ CH^4^/kg VS methane was produced from steam-exploded wheat straw after 215 and 650 days of digestion. Moreover, the methane potential of the untreated wheat straw in the present study was slightly lower than that in the previous studies [39, 40]. While studying the methane and ethanol production potential of wheat straw, Joelsson et al. [11] observed a methane yield potential of 0.321 Nm^3^/kg VS, from 1% acetic acid impregnated and steam-pretreated (190 to 210 °C) wheat straw. While using the fungal fermented wheat straw (Fig. 4, process alternative b), such as syrup R1 and R2, methane production potential of 0.25 ± 0.017 and 0.20 ± 0.005 Nm^3^ CH_4_/kg VS, respectively (Fig. 5), was observed. Moreover, with the co-digestion of thin stillage (from first-generation ethanol process) with R1 or R2 syrup (fungal fermented wheat straw) (Fig. 4, process alternative c), an increased methane production by about 14.7 or 6.1%, respectively, was observed as compared to that using only syrup R1 or R2. Anaerobic digestion of thin stillage alone led to a production of 0.22 ± 0.030 Nm^3^/kg VS methane under optimum conditions.

### Integrated Process Efficiency and Energy Output

Three different process scenarios are described in the present study for the biogas process (Fig. 4). Accordingly, (a) the pretreated wheat straw at two different conditions (as described in Validation of Dilute Acid Pretreatment at the Biorefinery Demonstration Plant section), (b) ethanol-separated fungal fermentation slurry (designated as syrup), and (c) thin stillage from first-generation starch-based ethanol process were used either directly or in combinations for the anaerobic digestion process. In general, all process streams connected to R1 pretreatment of straw gave higher methane yields (Fig. 5). Higher acid addition during the pretreatment, i.e., R1 conditions (1.2% acid, 195–197 °C), led to slightly higher concentrations of sugars (Table 1) in the pretreated slurry compared to those of R2 conditions (0.7% acid, 201–204 °C). The presence of higher amounts of inhibitors in the R1 hydrolysate however did not affect the biogas system, which is much diluted as opposed to the conditions used for fermentation and fungal cultivation [38]. Nevertheless, inhibitory substances (about 0.12 ± 0.002 g/g dry substrate wheat straw) resulting from the harsher pretreatment conditions (R1) most likely affected the ethanol production but not the biogas production, since both ethanol and biomass productions were lower after R1 treatment (Fig. 3). On the other hand, there were more fermentable sugars (about 0.42 ± 0.014 g/g dry substrate straw) remaining in the waste streams coming from the process, which, in turn, were available for biogas production, leading to about the same amount of methane (Fig. 5) from both the syrup (process alternative b on Fig. 4) and from the co-digestion of thin stillage and syrup mixture (process alternatives b and c on Fig. 4). Hence, while targeting biogas production, R1 treatment conditions are more beneficial, with the ethanol production step being excluded at times. After R1 treatment, almost three times more biogas can be produced from the treated straw (0.30 ± 0.033 Nm^3^ CH_4_/kg VS) corresponding to an increase by about 162%, compared to the methane potential of untreated straw (0.12 ± 0.024 Nm^3^ CH_4_/kg VS). Additionally, only a small difference (i.e., an increase by about 7.4% for R1) between the methane yields of the straw samples treated under the different conditions was observed (Fig. 5). Hence, the choice for the treatment conditions can be determined by comparing the energy demand needed for the treatment and the surplus energy produced in the form of improved methane production due to the treatment. At the optimum conditions using R1 and R2 pretreated slurry, an energy equivalent of about 16.8 and 15.7 MJ/kg dry wheat straw (higher heating values) was observed in the form of methane from the anaerobic digestion process, with about 161 and 144% improvement in the energy values of the untreated straw. On the other hand, the pretreatment with mild acid concentration (R2) is preferable for the integrated ethanol and biogas processes. With an ethanol yield of 0.16 kg and methane yield potential of 0.20 ± 0.005 Nm^3^ (per kg substrate and high heating values) from R2 hydrolysate and syrup, respectively, a combined energy output of 15.85 MJ/kg was obtained in this case. This corresponds to an increase in the energy output by about 10%, considering the heat generation potential of the untreated straw. In an integrated process of bioethanol, biohydrogen, and biogas production process, Kaparaju et al. [1] has reported an energy output from the biogas production of hydrothermal pretreated (at 180 °C for 15 min) wheat straw which was approximately 10% higher than that of untreated wheat straw.

Considering an integrated process scenario, the thin stillage and syrup (straw hydrolysate post the fungal cultivation) are co-digested; a slightly higher methane production can be achieved compared to the process where only the thin stillage is used in anaerobic digestion (Fig. 5). While using thin stillage and syrup R1 and R2 as the biogas substrate separately, an overall energy output of 12.4, 13.7, and 10.9 MJ/kg, respectively, was obtained. Whereas at the integrated process using the mixture of thin stillage and syrup R1 or R2, about 3.6 or 19.7%, respectively, increase in the energy output was observed compared to those obtained using the syrup R1 and R2 alone (Fig. 5). Hence, together with the ethanol production from the pretreated wheat straw, the integration of thin stillage for utilization in the biogas process can result in about 27 or 14% increase in the energy output from syrup R1 or R2, respectively (Figs. 3 and 5). In similar research studies for example, while using steam-pretreated oat straw to produce ethanol and biogas, Dererie et al. [41] reported an energy output of 9.5–9.8 MJ/kg dry straw, calculated from the amounts of ethanol and biogas produced. Similar energy output yields of 9.2 and 9.8 MJ/kg from corn stover pretreated with 0.2% sulfuric acid together with different combinations of pretreated slurry and thin stillage preparations had been reported previously [14].

Furthermore, an additional economic value is the *N. intermedia* fungal biomass obtained in the present study, with the crude protein content of 516 ± 34 mg/g dry fungal biomass, which could potentially be used as animal/fish feed [42]. Considering its protein, amino acid, lipid, and fatty acid composition as well as its high amino acid profile*, N. intermedia* biomass is a potential source of high-quality nutrients for feed applications [35]. The US Grains Council has announced an increased export of feed grains, reaching 100 million metric tons during 2015–2016 (http://www.grains.org). However, the demand for alternate protein concentrates within feeds is projected to exceed at a high rate, such as, for example, soymeal (at the price of 438 USD/ton) at about 2.8 million tons by 2020 [43]. Hence, there is a clear market potential for the use of an alternate protein source, such as the edible fungal biomass, produced in this integrated process, as an animal feed component. Moreover, the fungal biomass could also replace part of the fishmeal or soybean meal or to be added as an extract in the aquatic feed [22]. Thus, the current integrated process could potentially make new revenue streams for the existing wheat-based ethanol facilities in terms of lignocellulose (straw) based biofuels, i.e., ethanol and biogas, as well as fungal biomass for feed applications.

## Conclusions

In this study, different process scenarios are described for the integrated biorefinery model for ethanol, biogas, and edible fungi-based animal feed production. Accordingly, at the optimum conditions, the integrated second-generation process line results in about 90% of the theoretical maximum ethanol (based on the glucan content of the substrate wheat straw) and about 1.53 to 3.71 g/L dry fungal biomass, together with the methane yield potential in the range of 0.19 to 0.30 Nm^3^ CH_4_/kg VS (depending on the varying combinations of waste streams utilized for biogas production). The best overall energy output was obtained from wheat straw treated at higher acid concentrations as it resulted in a methane yield, by about 162% higher as compared to that of untreated straw. However, a detailed study on the techno-economic analysis of the different integrated process scenarios (which was not discussed in the present study) is also required for industrial applications and hence is open for the future studies.
